# Two new species of the genus *Xanthochlorus* from China (Diptera, Dolichopodidae, Xanthochlorinae)

**DOI:** 10.3897/zookeys.750.23382

**Published:** 2018-04-18

**Authors:** Qilemoge, Wencheng Chang, Ding Yang

**Affiliations:** 1 Department of Entomology, College of Plant Protection, China Agricultural University, Beijing 100193, China; 2 Shanghai Agricultural Technology Extension and Service Centre, Shanghai 201103, China

**Keywords:** China, Diptera, Dolichopodidae, new species, Xanthochlorinae, *Xanthochlorus*

## Abstract

The subfamily Xanthochlorinae comprises a single genus *Xanthochlorus*, and is rare in collections. Previously, there were four known species in the genus *Xanthochlorus* from China. In this paper, the species of the *Xanthochlorus* from China are reviewed. The following two species from Gansu Province of China are described as new to science: *Xanthochlorus gansuensis* **sp. n.** and *Xanthochlorus tewoensis* **sp. n.** A key to species of the *Xanthochlorus* from China is provided.

## Introduction

The subfamily Xanthochlorinae comprises only the single genus *Xanthochlorus* Loew. Compared to the other subfamilies of Dolichopodidae, most of which have high diversity, the genus *Xanthochlorus* has a low species number. There are presently 18 known species of *Xanthochlorus* in the world (Yang et al. 2006, 2011, Grichanov 2017), with only four species known in China: *Xanthochlorus chinensis* Yang & Saigusa, *X. nigricilius* Olejníček, *X. henanensis* Wang, Yang & Grootaert and *X. tibetensis* Xi, Wang & Yang (Olejníček 2004, Yang and Saigusa 2005, Wang et al. 2008, Yang et al. 2011, Xi et al. 2015). They have been reported from Shaanxi, Henan, and Tibet, which are, respectively, in the northwest and middle of China.

Gansu Province (32°31'–42°57'N, 92°13'–108°46'E) is located in the northwest of China and lies between the Tibetan Plateau and the Loess Plateau. The topography here is quite complicated, with mountains, basins, and deserts intersecting each other, which consequently also brings a complexity of the climates to this region. Due to a lack of precipitation, the northern part of Gansu province is dry and harsh all year round. However, the climate in the south of Gansu province is wet and mild, which leads to an abundant vegetation. These various environments contribute to the high species diversity of Gansu province. However, the investigation of the long-legged fly fauna here is still underway. In this paper two new species of the *Xanthochlorus* from Gansu province of China are reported and a key to the males of *Xanthochlorus* in China is provided. This is also the first time *Xanthochlorus* has been reported from Gansu province.

## Materials and methods

The specimens upon which this study is based, were collected from Gansu province of China in 2015 by sweeping nets. All specimens are deposited in the Entomological Museum of China Agricultural University (**CAU**), Beijing. Morphological terminology for adult structures mainly follows McAlpine (1981). Terms for the structures of the male genitalia follow Cumming and Wood (2009). The following abbreviations are used: **oc** = ocellar bristle (s), **vt** = vertical bristle (s), **pvt** = postvertical bristle (s), **acr** = acrostichal bristle (s), **ad** = anterodorsal bristle (s), **av** = anteroventral bristle (s), **dc** = dorsocentral bristle (s), **h** = humeral bristle (s), **npl** = notopleural bristle (s), **pa** = postalar bristle (s), **pd** = posterodorsal bristle (s), **ph** = posthumeral bristle (s), **sa** = supraalar bristle (s), **sc** = scutellars, **LI** = fore leg, **LII** = mid leg, **LIII** = hind leg. **CuAx ratio** = length of m-cu / length of distal portion of CuA, **hyp** = hypandrium, **epn** = epandrium, **sur** = surstylus, **cer** = cercus.

## Taxonomy

### Key to species (males) of *Xanthochlorus* from China

**Table:** 

1	Mid-posterior area of mesonotum and mid-basal area of scutellum blackish; CuAx less than 0.47	**2**
–	Mid-posterior area of mesonotum and mid-basal area of scutellum yellow, without blackish area; CuAx equal to 0.47	***X. tewoensis* sp. n.**
2	First flagellomere nearly quadrate (Fig. 4); cercus not serrated apically	**3**
–	First flagellomere semicircular; cercus serrated apically	***Xanthochlorus chinensis* Yang & Saigusa**
3	First flagellomere blunt apically; acr absent	**4**
–	First flagellomere with acute apical corner (Yang et al. 2011: p 1540, fig 988a); 3–4 acr	***X. henanensis* Wang, Yang & Grootaert**
4	Squama with brown hairs; hypandrium long, without short strip-like lateral protuberance	**5**
–	Squama with black hairs; hypandrium short, with short strip-like lateral protuberance (Figs 8–9)	***X. gansuensis* sp. n.**
5	Arista brown; hypandrium basally with short hook-like lateral protuberance	***X. tibetensis* Xi, Wang & Yang**
–	Arista yellow; hypandrium basally without short hook-like lateral protuberance	***X. nigricilius* Olejníček**

#### Xanthochlorus

Taxon classificationAnimaliaDipteraDolichopodidae

Loew, 1857

Xanthochlorus Loew, 1857: 42. Type species: Leptopus ornatus Haliday, 1832.

Diagnosis

Thorax and abdomen mainly yellow. Face narrower than frons, vertex flat, pvt absent. Antennal scape without hair, first flagellomere wider than long. Mid-posterior area of mesonotum flat. Mid and hind femora without preapical bristles. CuAx ratio 0.35–0.47. Male genitalia rather large and mostly exposed; cercus various, apically with hairs.

#### Xanthochlorus chinensis

Taxon classificationAnimaliaDipteraDolichopodidae

Yang & Saigusa, 2005

Xanthochlorus chinensis Yang & Saigusa, 2005: 754. Type locality: China, Shaanxi.

##### Diagnosis.

First flagellomere semicircular, distinctly wider than long (Yang et al. 2011: p 1539, fig 987a). Dorsal lobe of surstylus acute and curved apically, ventral lobe of surstylus irregularly furcated apically; cercus serrated apically; hypandrium with long lateral protuberance, strongly curved apically (Yang et al. 2011: p 1539, fig 987b).

##### Distribution.

Palaearctic: China (Shaanxi).

#### Xanthochlorus tewoensis sp. n.

Taxon classificationAnimaliaDipteraDolichopodidae

http://zoobank.org/3F0FA83D-7185-4A83-B8F7-6AEFF946C077

Figs 1 , 4–6

##### Diagnosis.

First flagellomere nearly quadrate. Mid-posterior area of mesonotum yellow. Mid-basal area of scutellum yellow. 1^st^ to 4^th^ tergites of abdomen pale yellow. Dorsal lobe of surstylus wide basally and sharp towards tip, with one bristle apically; ventral lobe of surstylus wide basally, with two apical protuberances; cercus small, nearly quadrate, weakly concave at middle dorsally; hypandrium straight, round apically.

##### Description.

**Figures 1–3.**
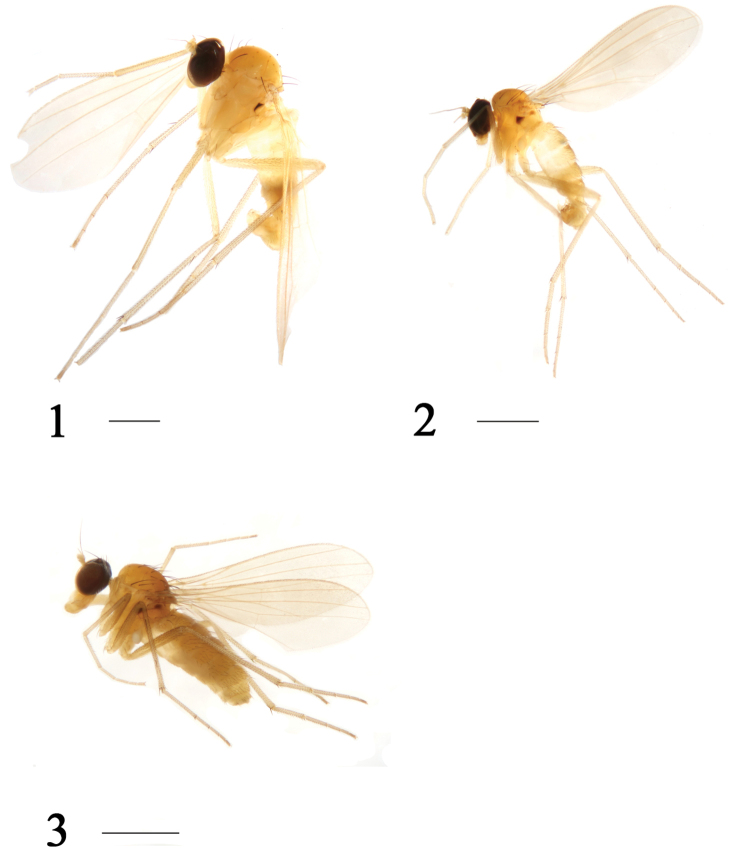
Habitus, lateral view. **1** *Xanthochlorus tewoensis* sp. n. Male. **2** *Xanthochlorus gansuensis* sp. n. Male. **3** *Xanthochlorus gansuensis* sp. n. Female. Scale bars: 1 mm.

**Figures 4–6.**
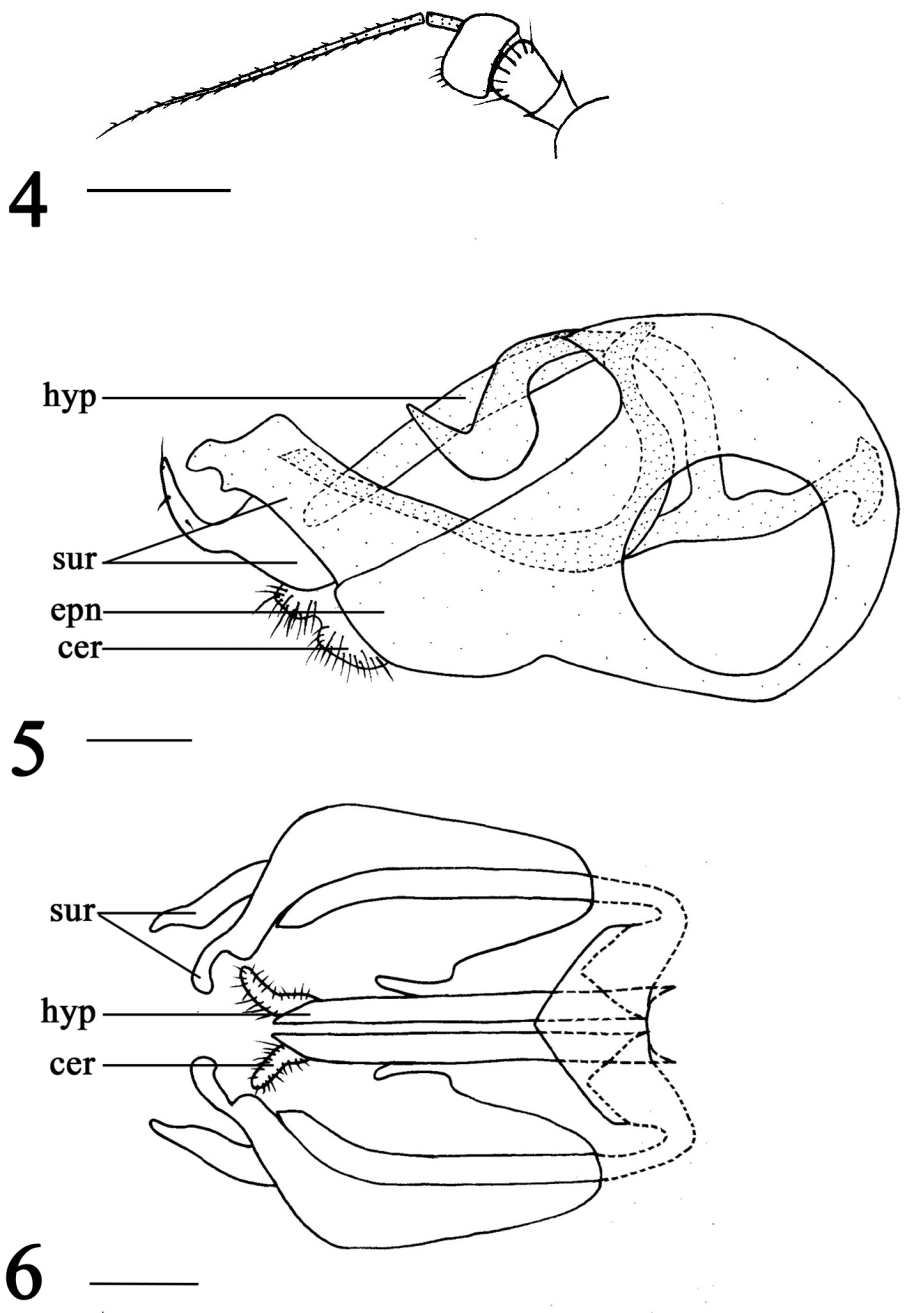
*Xanthochlorus tewoensis* sp. n., male. **4** Antenna **5** Genitalia, lateral view **6** Genitalia, ventral view. Abbreviations: hyp = hypandrium, epn = epandrium, sur = surstylus, cer = cercus. Scale bars: 0.2 mm.

Male (Fig. 1). Body length 3.0–3.2 mm. Wing length 3.3–3.8 mm. Head: metallic green with pale gray pollen. Hairs and bristles on head black. Face brown with pale gray pollen, width of face equal to length of first flagellomere. Postocular bristles all yellow. Two oc, two vt. Antenna (Fig. 4) yellow; first flagellomere nearly quadrate, 0.57 times as long as wide; arista brown, basal segment 0.09 times as long as apical segment. Proboscis yellow with yellow hairs; palpus yellow with yellow hairs.

**Figures 7–9.**
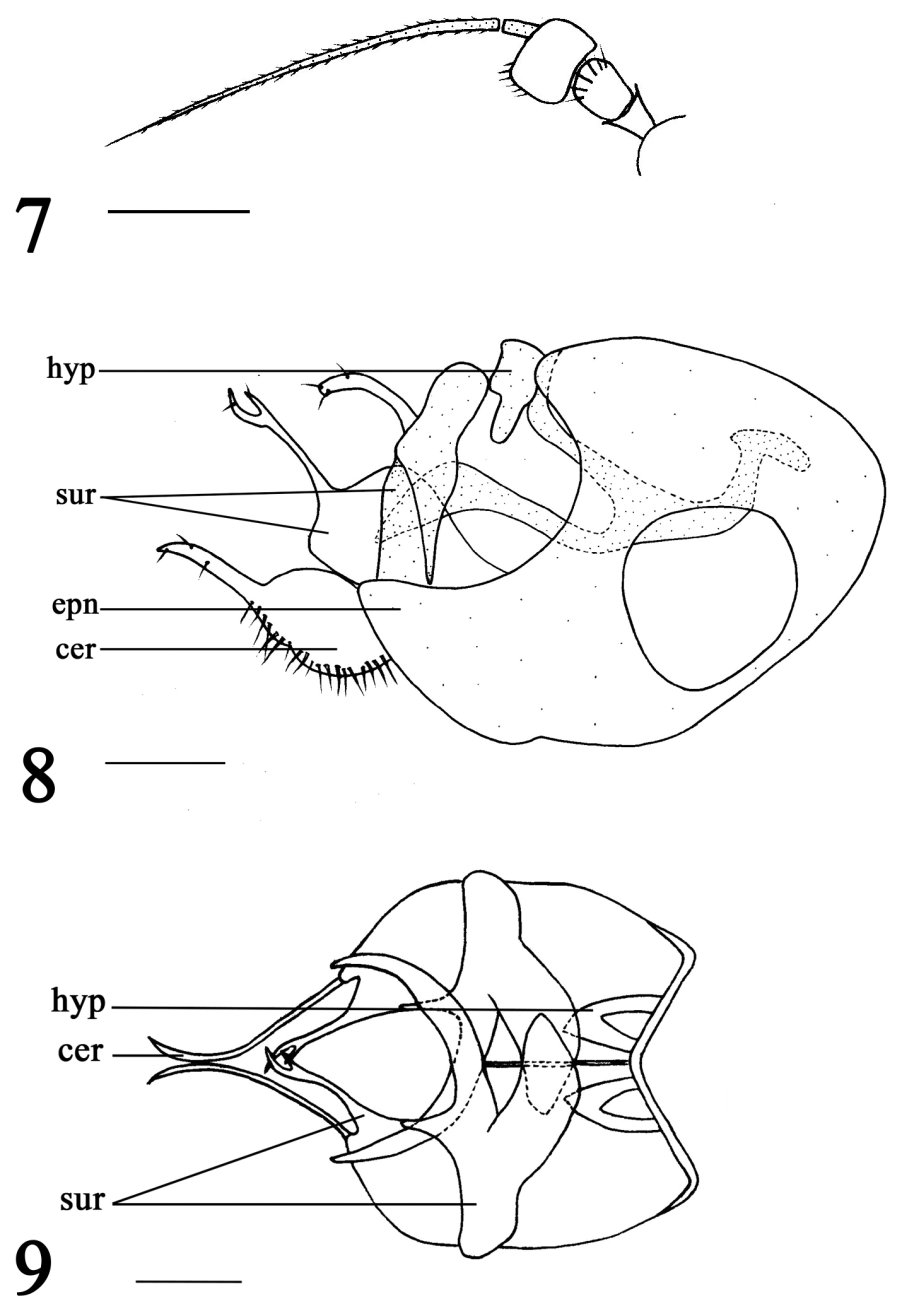
*Xanthochlorus gansuensis* sp. n., male. **7** Antenna **8** Genitalia, lateral view **9** Genitalia, ventral view. Abbreviations: hyp = hypandrium, epn = epandrium, sur = surstylus, cer = cercus. Scale bars: 0.2 mm.

Thorax yellow with pale gray pollen. Hairs and bristles on thorax black. Pteropleuron and latero-tergite each with a single black spot. Five strong dc, acr absent, one strong h, two strong npl, one strong su, one strong prsu, one strong sa, one strong pa; scutellum with a pair of sc. Propleuron with white hairs and a long yellow bristle on lower portion. Legs yellow. Hairs and bristles on legs black. Fore coxa with six bristles; middle and hind coxae each with a single black outer bristle. Fore tibia without distinct bristle; middle tibia with one ad, one pd, and four apical bristles; hind tibia with two apical bristles. Mid and hind femora without preapical bristles. Relative lengths of tibia and 5 tarsomeres LI 6.0 : 4.0 : 1.6 : 1.2 : 0.8 : 0.7; LII 6.0 : 4.0 : 1.6 : 1.2 : 0.8 : 0.7; LIII 9.4 : 2.8 : 1.9 : 1.2 : 0.8 : 0.6. Wing hyaline, veins brown; costal callus indistinct; M gently bent apically, M and R_4+5_ parallel apically; CuAx ratio 0.47. Squama yellow with yellow hairs. Halter yellow.

Abdomen yellow with pale gray pollen, tergites 1–4 pale yellow. Hairs and bristles on abdomen black. Male genitalia (Figs 4–6): Epandrium longer than wide; dorsal lobe of surstylus yellow, basally wide, nearly quadrate, apically sharp with one bristle; ventral lobe of surstylus pale brown, wide, apically with two protuberances; cercus small, nearly quadrate with distinct bristles, but weakly concave at middle dorsally; hypandrium light brown, straight, round apically.

Female. Unknown.

##### Types.

Holotype male, CHINA, Gansu, Tewo, Lazikoulinchang, Laolazi, collected by sweeping nets in grassland, 2015.VII.26, Qingxia Zhou (CAU). Paratypes: two males, collecting information same as the holotype (CAU).

##### Distribution.

Palaearctic: China (Southern wet part of Gansu).

##### Remarks.

The new species is somewhat similar to *X. chinensis* Yang and Saigusa, but can be separated from the latter by the quadrate first flagellomere, the yellow mid-posterior area of the mesonotum and the two protuberances on the ventral lobe of the surstylus. In *X. chinensis*, the first flagellomere is semicircular, the mid-posterior area of the mesonotum is black, and the ventral lobe of the surstylus is irregularly furcated at tip (Yang and Saigusa 2005, Yang et al. 2011: p 1539, fig 987b).

##### Etymology.

The specific name refers to the type locality of Tewo.

#### Xanthochlorus gansuensis sp. n.

Taxon classificationAnimaliaDipteraDolichopodidae

http://zoobank.org/2274D456-2FB4-452F-AD14-69DB88178347

Figs 2–3 , 7–9

##### Diagnosis.

First flagellomere nearly quadrate. Mid-posterior area of mesonotum blackish. Mid-basal area of scutellum blackish. Dorsal lobe of surstylus wide basally, nearly quadrate, apically concaved with two bristles; ventral lobe of surstylus wide basally, band-like, apically rounded with two bristles; cercus ovate basally, apically finger-like with bristles; hypandrium rather short with short strip-like lateral protuberance.

##### Description.

Male (Fig. 2). Body length 2.3–2.6 mm. Wing length 2.6–2.9 mm. Head: metallic green with pale gray pollen. Hairs and bristles on head black. Face brown with pale gray pollen, width of face equal to length of first flagellomere. Postocular bristles all yellow.Two oc, two vt. Antenna (Fig. 7) yellow; first flagellomere nearly quadrate, 0.6 times as long as wide; arista pale brown, basal segment 0.1 times as long as apical segment. Proboscis yellow with pale brown hairs; palpus yellow with pale brown hairs.

Thorax yellow with pale gray pollen. Hairs and bristles on thorax black. Mid-posterior area of mesonotum blackish; mid-basal area of scutellum blackish; pteropleuron and latero-tergite each with one black spot. 5 strong dc, acr absent, one strong h, two strong npl, one strong su, one strong prsu, one strong sa, one strong pa; scutellum with one pair of sc. Propleuron with white hairs and one long yellow bristle on lower portion. Legs yellow. Hairs and bristles on legs black. Fore coxae with 5–6 bristles; middle and hind coxae each with one outer bristle. Fore tibia without distinct bristles; middle tibia with two ad, one pd, and four apical bristles; hind tibia with three apical bristles. Mid and hind femora without preapical bristle. Relative lengths of tibia and 5 tarsomeres LI 3.0 : 1.5 : 0.8 : 0.6 : 0.4 : 0.4; LII 3.8 : 1.7 : 0.7 : 0.5 : 0.4 : 0.4; LIII 4.5 : 1.4 : 1.0 : 0.7 : 0.4 : 0.4. Wing hyaline, veins brown; costal callus indistinct; M gently bent apically, M and R_4+5_ parallel apically; CuAx ratio 0.38. Squama yellow with black hairs. Halter yellow.

Abdomen yellow with pale gray pollen. Hairs and bristles on abdomen black. Male genitalia (Figs 7–9): Epandrium longer than wide; dorsal lobe of surstylus yellow, wide basally, nearly quadrate, apically concaved with two bristles; ventral lobe of surstylus pale brown, wide basally, band-like, apically rounded with two bristles; cercus ovate basally, apically finger-like with bristles; hypandrium brown, rather short with short strip-like lateral protuberance.

Female (Fig. 3). Body length 2.6 –2.8 mm. Wing length 2.7–2.9 mm.

##### Types.

Holotype male, CHINA, Gansu, Tianshui, Dangchuan Linchang, Maicaogou; collected by sweeping nets in grassland, 2015.VII.17, Xiaoli Li (CAU). Paratypes: nine males, 13 females, collecting information same as the holotype (CAU).

##### Distribution.

Palaearctic: China (Southern wet part of Gansu).

##### Remarks.

The new species is somewhat similar to *X. henanensis* Wang, Yang and Grootaert, but can be separated from the latter by the first flagellomere apically without acute corner and by the lack of acrostichals. In *X. henanensis*, the first flagellomere has an acute lower apical corner and 3–4 acrostichals are present (Wang et al. 2008, Yang et al. 2011: p 1540, fig 988a).

##### Etymology.

The specific name refers to the type locality Gansu.

#### Xanthochlorus henanensis

Taxon classificationAnimaliaDipteraDolichopodidae

Wang, Yang & Grootaert, 2008

Xanthochlorus henanensis Wang, Yang & Grootaert, 2008: 253. Type locality: China, Henan.

##### Diagnosis.

First flagellomere slightly wider than long, nearly quadrate, acute apically (Yang et al. 2011: p 1540, fig 988a). Mesonotum with 3–4 acrostichals. Scutellum metallic green with brownish margin. Mid tarsomere one longer than tarsomeres 2–5 combined, with two rows of ventral bristles. Dorsal lobe of surstylus long and wide, apically hook-like, ventral lobe of surstylus short, furcated apically; cercus nearly rounded; hypandrium with short lateral protuberance, irregularly furcated apically (Yang et al. 2011: p 1540, fig 988b).

##### Distribution.

Palaearctic: China (Henan).

#### Xanthochlorus nigricilius

Taxon classificationAnimaliaDipteraDolichopodidae

Olejníček, 2004

Xanthochlorus nigricilius Olejníček, 2004: 9. Type locality: China, Shaanxi.

##### Diagnosis.

Antenna and arista wholly yellow. First flagellomere as long as wide, nearly quadrate (Olejníček 2004: p 10, fig 1). Mid-posterior area of mesonotum and basal area of scutellum black. Dorsal lobe of surstylus apically acute and strongly curved (Olejníček 2004: p 10, figs 2–3).

##### Distribution.

Palaearctic: China (Shaanxi).

#### Xanthochlorus tibetensis

Taxon classificationAnimaliaDipteraDolichopodidae

Xi, Wang & Yang, 2015

Xanthochlorus tibetensis Xi, Wang & Yang, 2015: 315. Type locality: China, Tibet.

##### Diagnosis.

Bristles on head mostly yellow, but those on thorax black. First flagellomere nearly quadrate. Mid tarsomere one with two short weak av. Dorsal surstylus acute and curved apically, ventral surstylus wide, furcated apically; cercus bent, wide basally and finger-like apically; hypandrium basally with short hook-like lateral protuberance, apically deeply incised with lateral protuberance, slightly curved (Xi et al. 2015: p 315, fig 5).

##### Distribution.

Palaearctic: China (Tibet).

## Supplementary Material

XML Treatment for Xanthochlorus

XML Treatment for Xanthochlorus chinensis

XML Treatment for Xanthochlorus tewoensis

XML Treatment for Xanthochlorus gansuensis

XML Treatment for Xanthochlorus henanensis

XML Treatment for Xanthochlorus nigricilius

XML Treatment for Xanthochlorus tibetensis
